# Calvarial melorheostosis: an extremely rare case and diagnostic challenge

**DOI:** 10.1007/s00256-025-04882-w

**Published:** 2025-02-13

**Authors:** Gregory Wenokor, David Suster, Ada Baisre de Leon, James K. Liu, Cornelia Wenokor, Esther A. Nimchinsky

**Affiliations:** 1 Department of Radiology, Rutgers New Jersey Medical School, MSB F-506, 185 South Orange Avenue, Newark, NJ 07103 USA; 2 Department of Pathology, Immunology and Laboratory Medicine, Rutgers New Jersey Medical School, MSB C-579, 185 South Orange Avenue, Newark, NJ 07103 USA; 3 Department of Neurosurgery, Cooperman Barnabas Medical Center, RWJ Barnabas Health, Livingston, NJ 07039 USA; 4 Skull Base Institute of New Jersey, Neurosurgeons of New Jersey, NYU Langone Neurosurgery Network, Livingston, NJ 07039 USA

**Keywords:** Melorheostosis, Leri’s disease, Calvarium

## Abstract

Melorheostosis is a rare bone disease that presents as sclerotic lesions growing on existing bone surfaces. This disease is of unknown etiology and affects both genders equally. The disease is unique in appearance, described in the literature as having a radiologic appearance of flowing candle wax, and usually occurs in the appendicular skeleton. We present an extremely rare case of melorheostosis of the calvarium in a 55-year-old male patient, initially thought to represent a large osteoma, and discuss the CT and MRI features, pathology, and surgical management. Recently, new insights have been gained by molecular biologic studies into the potential pathogenesis of this disease indicating genetic mutations, which will be discussed. This case report will help radiologists arriving at the correct diagnosis for an unusual manifestation of a rare disease.

**Supplementary Information:**

The online version contains supplementary material available at 10.1007/s00256-025-04882-w.

## Introduction

Melorheostosis (Leri’s disease) is a rare, non-inheritable, sclerosing bone disease characterized by excessive bone growth on existing bone surfaces. While it is a benign process, the bone growth can cause symptoms such as chronic pain, joint deformation, and limited range of motion in the affected area [1, 2]. The diagnosis is usually made by the radiologic identification of “flowing” osteosis, whose appearance is often compared to dripping candle wax. However, other presentations can occur that do not have the same appearance [3]. These may appear similar to osteoma, myositis ossificans, and osteopathia striata or have mixed appearances [3]. Melorheostosis most frequently involves the appendicular skeleton. However, the axial skeleton is affected in some cases, and the process can be either monostotic or polyostotic [4]. Craniofacial involvement is rare and usually involves the facial bones [5]. Only one clear case of calvarial melorheostosis has been reported to date [6], and this case primarily involved the facial bones with minor involvement of the calvarium. We present an extremely rare case of melorheostosis of the calvarium with an osteoma-like appearance. The patient gave consent for publication of this study.

## Case report

**Fig. 1:**
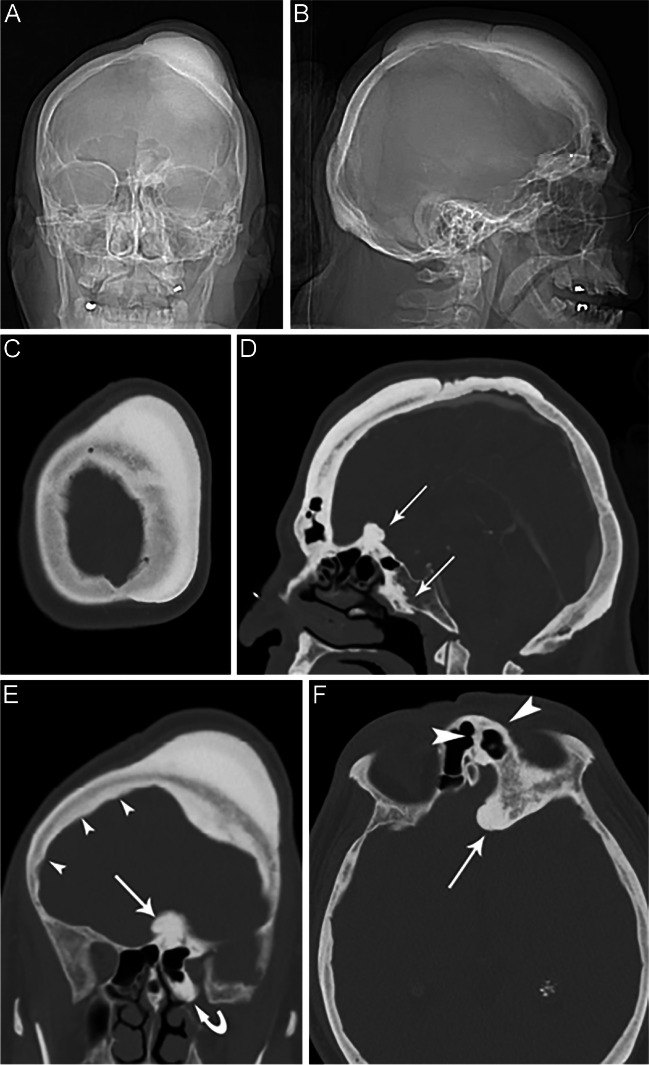
CT angiogram of the head, performed for preoperative planning. Scout images: AP view (**A**) and lateral view (**B**). Hyperostotic bone involves the left calvarium in the frontal and parietal region, mainly superficial to the outer cortex. **C** Axial image showing a thick rind of cortical bone layering on the outer cortex of the left parietal bone. **D** Sagittal image demonstrating the marked cortical thickening along the frontal bone and involving the sphenoid bone, extending inferiorly along the anterior clivus (arrows). **E** Coronal image shows the extension of cortical thickening deep to the inner cortex, as well as the involvement of the sphenoid bone and extending distally along the left fovea ethmoidalis and planum sphenoidale. There was marked cortical thickening of the inner table extending into the right frontal bone (arrowheads) and a pedunculated hyperostotic lesion along the planum sphenoidale (arrow). **F** Axial image at the level of the orbits shows the sphenoid bone involvement and cortical thickening of the left frontal sinus (arrowheads) and the pedunculated lesion along the planum sphenoidale (arrow)

A 55-year-old Hispanic male with a past medical history of hypertension, hyperlipidemia, insulin-dependent diabetes, right eye vision loss, and peptic ulcer disease presented to the emergency department complaining of headache and swelling on the left side of his head. The patient stated that the swelling had been present for 20–30 years, and he now wanted the swelling treated. He denied any symptoms of dizziness, headache, diaphoresis, or chest pain. He was noted to have a palpable protrusion associated with the area of swelling and the patient stated that it was not associated with any particular symptoms. Routine lab tests were done, which were normal except for glucose, in keeping with his history of diabetes. Of note, alkaline phosphatase levels were normal at 46 IU/l, as was serum calcium of 8.8 mg/dl (normal 8.4–10.2 mg/dl). Parathyroid hormone levels were not obtained. As part of his operative planning for resection of the calvarial mass, he underwent a CT angiogram of the head. Scout films (Fig. 1A, B) showed a sclerotic bony lesion at the vertex involving both the left frontal and parietal bones, largely superficial to the outer table and displacing the overlying soft tissues. The absence of the typical flowing candle wax appearance characteristic of melorheostosis initially gave the impression of a large osteoma.

Computed tomography (CT) images (Fig. 1C–F) showed the growth as dense cortical thickening along both the inner and outer calvarial tables, with preservation of the normal diploic space. From the vertex, the lesion extended inferiorly to the left anterior cranial fossa. Sclerotic areas as well as areas of trabecular thickening were seen in the diploic space. The mass at the vertex measured 11.2 cm in its largest dimension, anteroposteriorly. A separate smaller pedunculated lesion was noted at the left planum sphenoidale, measuring 1.4 cm. The angiogram portion of this exam showed that all arteries were clear of stenosis or occlusion except for the cavernous portions of the right internal carotid artery, which had some atherosclerotic calcifications, but no evidence of significant stenosis (not shown). CT angiogram showed no evidence of arterial impingement by the mass. Notably, the mass did not extend to the groove for the middle meningeal artery.

**Fig. 2:**
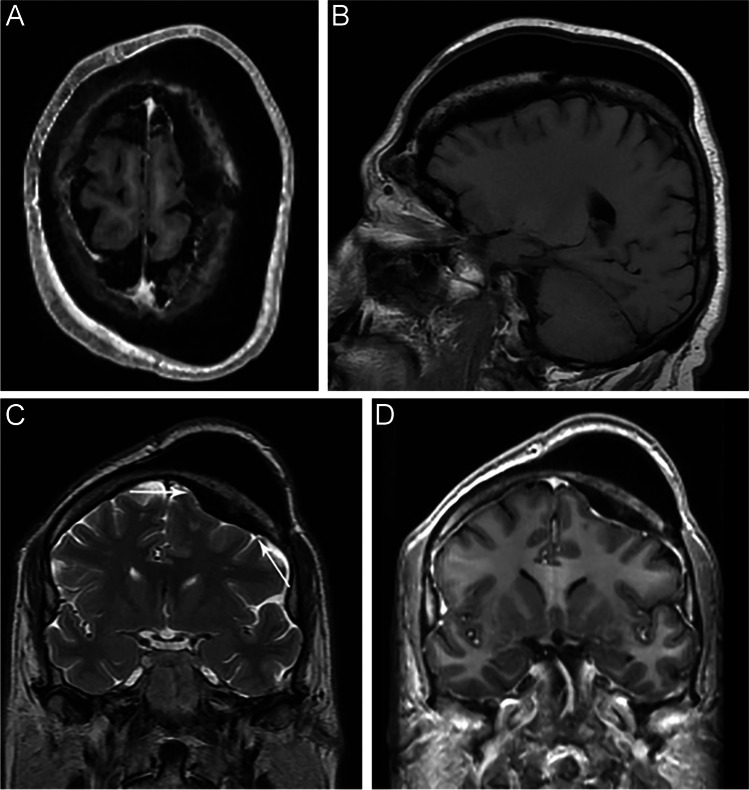
An MRI brain obtained 8 years earlier. Axial (**A**) and sagittal (**B**) T1 weighted turbo spin echo (TSE), coronal T2 weighted sequence (**C**), and coronal gadolinium-enhanced T1 weighted sequence (**D**). The massive cortical thickening has not significantly changed in appearance in the 8 years since this study was performed. As it is cortical bone, it appears black on all sequences, and there is no evidence of contrast enhancement

The radiologic differential diagnosis included osteoma, and the presence of the secondary lesion in the sphenoid raised concerns for Gardner syndrome. Metabolic bone disease as well as other hyperostotic syndromes such as Van Buchem’s disease were considered given the location; however, melorheostosis was favored due to the appearance of the lesion on the CT scans, as well as the lack of interval growth when compared to an MRI scan obtained 8 years prior (Fig. 2).

**Fig. 3:**
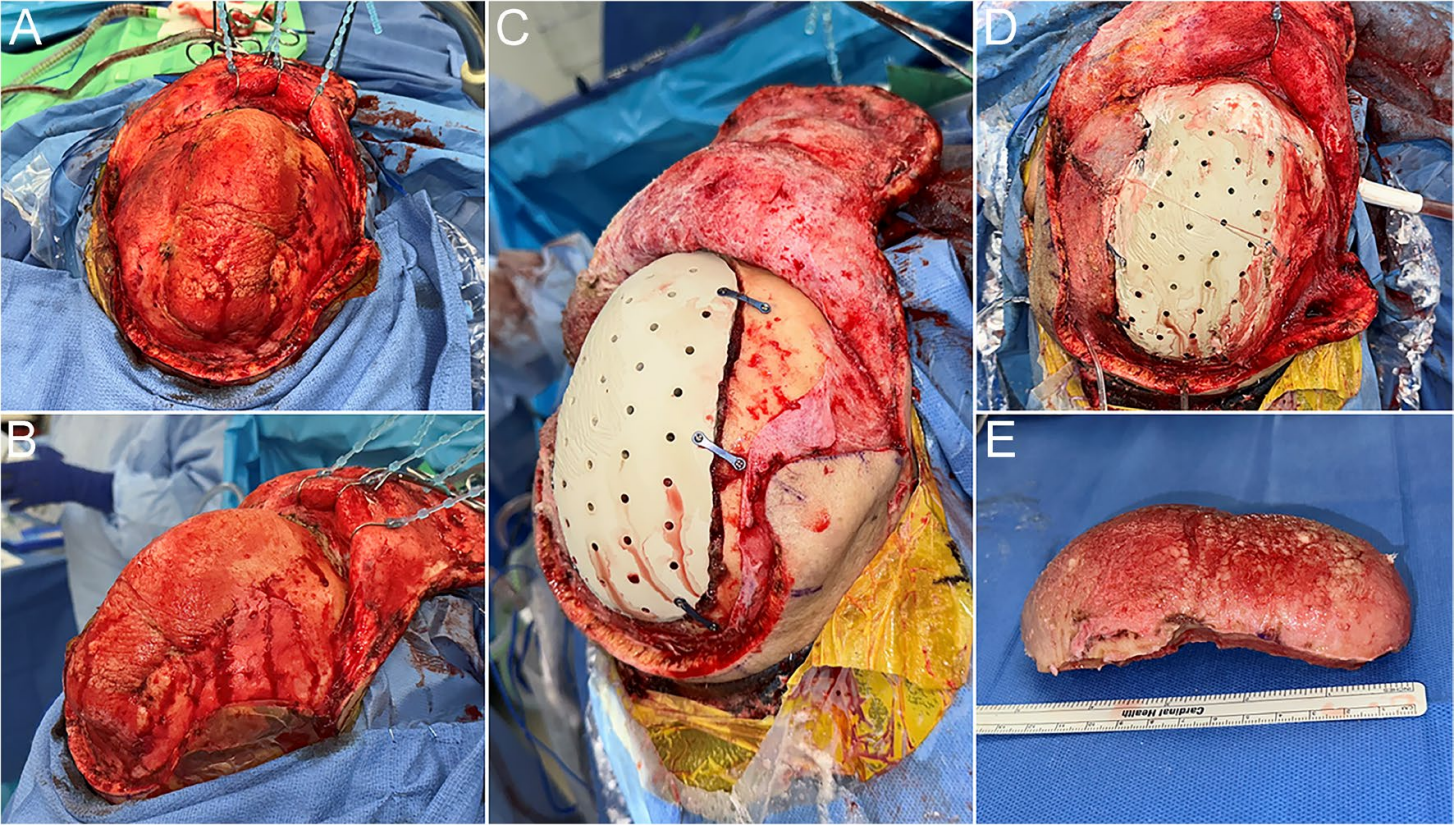
**A** and **B** Intraoperative view of calvarial mass. **C** and **D** After resection of the calvarial mass, the skull defect is reconstructed with a patient-specific PEEK custom implant. **E** Calvarial mass resected

**Fig. 4:**
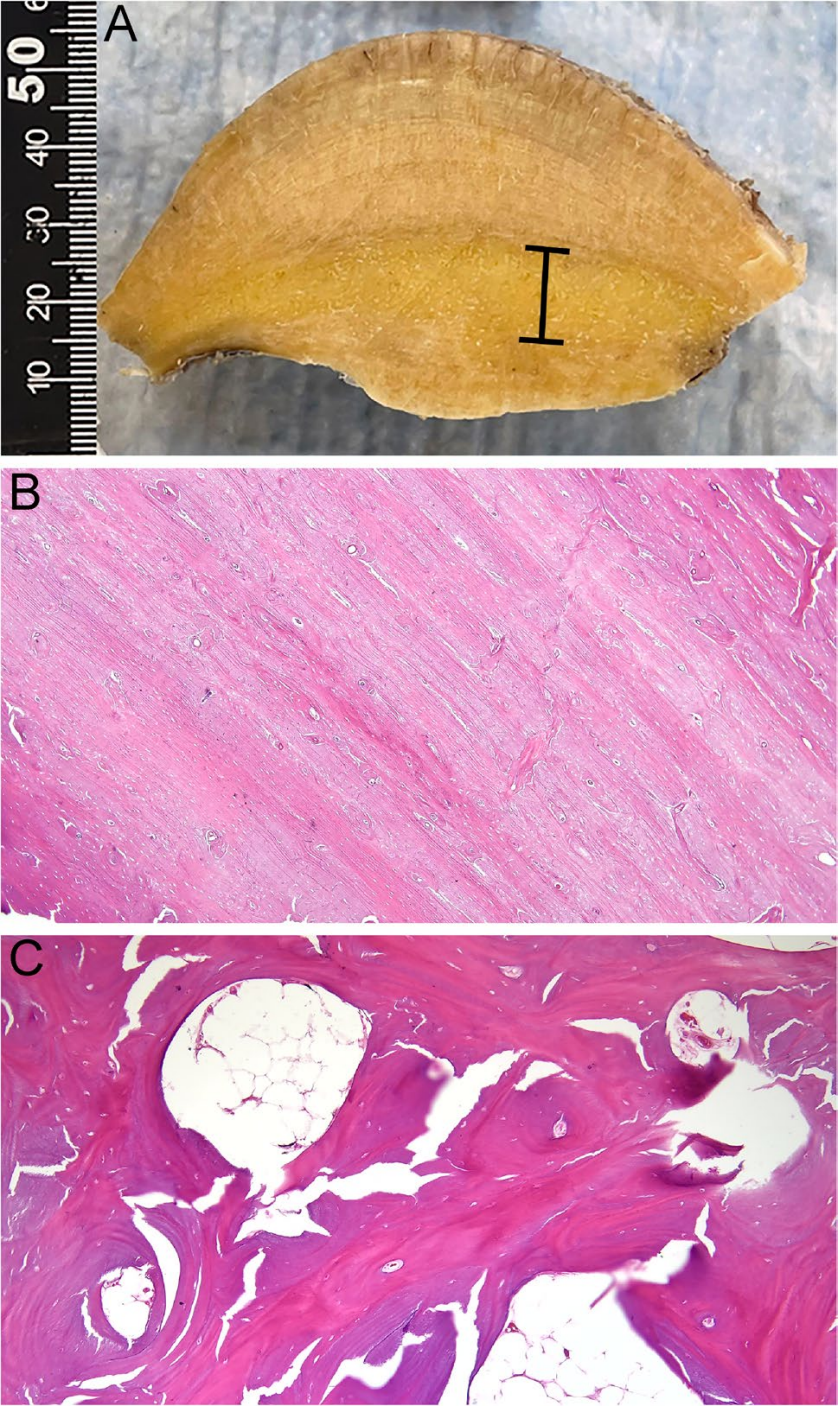
Pathology: gross and microscopic features. **A** Gross photograph demonstrates an expansion of the calvarium with layered sclerotic bone growth along both the outer and inner tables. The diploic space is indicated by the black bar. **B** Hematoxylin and eosin (H&E), 2 × magnification: Cut section from lesion shows dense sclerotic lamellar bone with an intact haversian canal system. **C** Hematoxylin and eosin (H&E), 4 × magnification: Low power magnification of cut section from lesion shows thickened bony trabeculae composed of lamellar bone with intervening fatty marrow

The patient underwent a left frontoparietal craniectomy for the resection of the calvarial lesion, a left orbitotomy for decompression of the orbital roof and resection of the smaller secondary lesion in the region of the anterior sphenoid, and a single-stage cranioplasty using a custom PEEK (polyetheretherketone) patient-specific 3D-printed custom cranial implant (Fig. 3, Supplemental Fig. 1). Pathologic examination of the primary mass showed a lesion grossly composed of dense sclerotic bone with growth along the outer and inner table of the calvarium (Fig. 4A). Histologic examination showed variable findings ranging from dense, sclerotic, lamellar bone (Fig. 4B), and areas of disorganized bone growth with several Haversian canals oriented perpendicularly to the appositional bone and fatty marrow in the intervening trabecular spaces with absence of a significant osteoblastic/osteoclastic component (Fig. 4C).

## Discussion

Melorheostosis is a rare mesenchymal bone dysplasia that presents as excessive sclerotic bone that grows on pre-existing host bone surfaces. It was first described in 1922 by Léri and Joanny [7]. Its name derives from the Greek “melos” for limb, “rhein” for flowing, and “ostosis” for bone formation [1]. Classically, the presentation of melorheostosis as described in literature is that of osteosis with the appearance of flowing wax. The disease usually begins sporadically in adolescence and has no sex predilection [8, 9]. Diagnosis of the disease usually occurs at ages 20 or younger [8–10].

As is evident from the name of the disorder, melorheostosis typically appears in the appendicular skeleton. Involvement of the axial skeleton is rare [11], and craniofacial involvement is even more so [12]. A case of craniofacial melorheostosis involving the facial bones and a portion of the calvarium was reported previously [6], but to our knowledge, no clear cases centered in the calvarium have been reported.

This case of melorheostosis did not have the flowing candle wax appearance first described by Léri and Joanny [7], presumably due to the vastly different configuration of the bone involved. Initial impressions of the radiographs and computed tomography studies led to a differential diagnosis including osteoma/Gardner syndrome and genetic or metabolic bone syndromes such as Van Buchem syndrome or hypercalcemia. The initial radiologic impression of an osteoma is reminiscent of some studies that show some cases of melorheostosis with an osteoma-like appearance [3, 13]. According to one series, the osteoma-like appearance is more common than the candle wax appearance; out of 23 cases, 7 (30%) had an osteoma-like appearance and 5 (22%) had the candle wax appearance [3]. In the present case, there were some areas of focal sclerosis radiographically within the diploic space, indicating that the process was not confined only to the outer and inner tables, further distinguishing this entity from osteoma. Diploic involvement is analogous to marrow involvement seen in melorheostosis in long bones, where bone marrow edema may also be seen [14]. In addition, osteomas are usually much smaller than the case presented and often are isolated lesions. Our patient also had no other signs or symptoms of Gardner syndrome, making this diagnosis less likely. Van Buchem syndrome is a heritable sclerosing bone dysplasia that results in extensive osteosis, and when present in the skull involves thickening of the maxilla and mandible [15]. However, it has almost entirely been recorded in people of Dutch descent, or from other nearby regions, making it less likely in this case.

The exact etiology of melorheostosis is unknown. Much of the research in melorheostosis has been in the context of coexistence with osteopoikilosis, Buschke-Ollendorff syndrome, and mixed sclerosing bone dysplasia [16]. While the cases that coexist with other diseases are much rarer than sporadic cases, it is of interest that overlap exists among these diseases. A loss-of-function mutation in the *LEMD3* gene has been described in osteopoikilosis and Buschke-Ollendorff syndrome. The MAN1 inner nuclear membrane protein encoded by *LEMD3* is responsible for the modulation of TGFβ and bone morphogenetic protein signaling, which activate genes involved in bone formation. Melorheostosis concurrent with osteopoikilosis has been shown to be associated with the *LEMD3* mutation [17]. Sporadic cases of melorheostosis are not associated with the loss-of-function mutation of *LEMD3*. Instead, it has been shown that the bone associated with melorheostosis has a mutation in the *MAP2K1* gene, which encodes the protein kinase MEK1 [16, 17]. The mutation results in a loss of function for MEK1, which in turn increases osteoblast proliferation and reduces their differentiation. The result of this mutation may explain the histologic features described in melorheostosis, an increase in osteoblast/osteoclast number as well as an increase in osteoid and bone remodeling. Interestingly, cases of melorheostosis in which the *MAP2K1* mutation is present are the cases in which the flowing candle wax appearance is noted [18] as well as the previously mentioned histologic findings. Cases of melorheostosis without the *MAP2K1* mutation lack the candle wax sign and do not have increased osteoblast/osteoclast counts when examined histologically. In our case, DNA sequencing was attempted; however, insufficient genomic material was available for genomic testing due to the nature of the tissue submitted.

While there is a reported quantitative difference in the counts of osteoblasts/osteoclasts between *MAP2K1* mutation positive and negative cases of melorheostosis, there are other features that may be useful in aiding the diagnosis of melorheostosis histologically. While these vary from case to case, these findings include an increase in cortical density, presence of woven bone, increased porosity, disorganized osteoid formation, and angiogenesis [4]. However, the presence of these features can be variable and in some cases present only with an increase in cortical density as in our case [4]. Histologic analysis of melorheostosis is not necessarily required for the diagnosis if the classic radiologic findings are present; however, in some cases, it is needed to rule out potential malignancy as some bone-forming tumors, such as parosteal osteosarcoma, may have similar radiologic characteristics [19]. By histology, these two entities can usually be separated by identifying the classic features of a parosteal osteosarcoma including long parallel arrays of bone with an infiltrating, bland, but atypical, spindle cell proliferation. Fluorescence in situ hybridization may also be of benefit as parosteal osteosarcoma is known to harbor amplification of the *MDM2* gene [20].

The diagnosis of melorheostosis is often suggested due to the radiologic recognition of the flowing candle wax appearance as described in the literature. However, there exist cases of melorheostosis that do not fit this profile, and careful radiologic and pathologic analysis can be essential in making this diagnosis.

## Supplementary Information

Below is the link to the electronic supplementary material.

Supplementary file1 (DOCX 369 KB)

## Data Availability

All data and materials are available for review from the authors upon reasonable written request.

## References

[1] Iordache S, Cursaru A, Serban B, Costache M, Spiridonica R, Cretu B, et al. Melorheostosis: a review of the literature and a case report. Medicina [Internet]. 2023;59. 10.3390/medicina59050869 10.3390/medicina59050869 PMC10223220 37241101

[2] Greenspan A, Azouz EM. Bone dysplasia series. Melorheostosis: review and update. Can Assoc Radiol J. 1999;50:324–30. 10555508

[3] Freyschmidt J. Melorheostosis: a review of 23 cases. Eur Radiol. 2001;11:474–9. 11288855 10.1007/s003300000562

[4] Fick CN, Fratzl-Zelman N, Roschger P, Klaushofer K, Jha S, Marini JC, et al. Melorheostosis: a clinical, pathologic, and radiologic case series. Am J Surg Pathol. 2019;43:1554–9. 31386640 10.1097/PAS.0000000000001310 PMC7832124

[5] McDermott M, Branstetter BF 4th, Seethala RR. Craniofacial melorheostosis. J Comput Assist Tomogr. 2008;32:825–7. 18830120 10.1097/RCT.0b013e3181572998

[6] Ethunandan M, Khosla N, Tilley E, Webb A. Melorheostosis involving the craniofacial skeleton. J Craniofac Surg. 2004;15:1062–5. 15547407 10.1097/00001665-200411000-00038

[7] Leri A, Joanny J. Une affection non decrite des os hyperostose “en coulee” sur toute la longeur d’un member ou “melorheostose.” Bull Mem Soc Med Hosp Paris. 1922;46:1141–5.

[8] Kumar R, Sankhala SS, Bijarnia I. Melorheostosis - Case Report of Rare Disease. J Orthop Case Rep. 2014;4:25–7. 27298954 10.13107/jocr.2250-0685.162 PMC4719368

[9] Vyskocil V, Koudela K Jr, Pavelka T, Stajdlova K, Suchy D. Incidentally diagnosed melorheostosis of upper limb: case report. BMC Musculoskelet Disord. 2015;16:2. 25637225 10.1186/s12891-015-0455-z PMC4320463

[10] Biaou O, Avimadje M, Guira O, Adjagba A, Zannou M, Hauzeur J-P. Melorheostosis with bilateral involvement in a black African patient. Joint Bone Spine. 2004;71:70–2. 14769526 10.1016/S1297-319X(03)00103-9

[11] Suresh S, Muthukumar T, Saifuddin A. Classical and unusual imaging appearances of melorheostosis. Clin Radiol. 2010;65:593–600. 20599060 10.1016/j.crad.2010.02.004

[12] Tueche SG, Gebhart M, Dewolf J, Baillon JM, Limbosch JM. Cranio-facial and humeral melorrheostosis. Acta Chir Belg. 1999;99:47–50. 10090966

[13] Jain VK, Arya RK, Bharadwaj M, Kumar S. Melorheostosis: clinicopathological features, diagnosis, and management. Orthopedics. 2009;32:512. 19634844 10.3928/01477447-20090527-20

[14] Hurley-Novatny A, Karantanas AH, Papadakis GZ, Bhattacharyya T, Jha S. Cross-sectional imaging useful in melorheostosis. JBMR Plus. 2021;5:e10472. 33869990 10.1002/jbm4.10472 PMC8046147

[15] Wengenroth M, Vasvari G, Federspil PA, Mair J, Schneider P, Stippich C. Case 150: Van Buchem disease (hyperostosis corticalis generalisata). Radiology. 2009;253:272–6. 19789259 10.1148/radiol.2531080011

[16] Wordsworth P, Chan M. Melorheostosis and osteopoikilosis: a review of clinical features and pathogenesis. Calcif Tissue Int. 2019;104:530–43. 30989250 10.1007/s00223-019-00543-y

[17] Kang H, Jha S, Deng Z, Fratzl-Zelman N, Cabral WA, Ivovic A, et al. Somatic activating mutations in MAP2K1 cause melorheostosis. Nat Commun. 2018;9:1390. 29643386 10.1038/s41467-018-03720-z PMC5895796

[18] Jha S, Fratzl-Zelman N, Roschger P, Papadakis GZ, Cowen EW, Kang H, et al. Distinct clinical and pathological features of melorheostosis associated with somatic MAP2K1 mutations. J Bone Miner Res. 2019;34:145–56. 30138550 10.1002/jbmr.3577 PMC7577747

[19] Brennan DD, Bruzzi JF, Thakore H, O’Keane JC, Eustace S. Osteosarcoma arising in a femur with melorheostosis and osteopathia striata. Skeletal Radiol. 2002;31:471–4. 12172596 10.1007/s00256-002-0495-y

[20] Dujardin F, Binh MBN, Bouvier C, Gomez-Brouchet A, Larousserie F, de Muret A, et al. MDM2 and CDK4 immunohistochemistry is a valuable tool in the differential diagnosis of low-grade osteosarcomas and other primary fibro-osseous lesions of the bone. Mod Pathol. 2011;24:624–37. 21336260 10.1038/modpathol.2010.229

