# Supplementary material for: Calvarial melorheostosis: an extremely rare case and diagnostic challenge
Source: Skeletal Radiol. 2025 Feb 13;54(8):1761–6. doi: 10.1007/s00256-025-04882-w (PMC12174187; doi:10.1007/s00256-025-04882-w)
Supplement: Supplementary file 1 — Supplementary file1 (DOCX 369 KB) [file 256_2025_4882_MOESM1_ESM.docx]

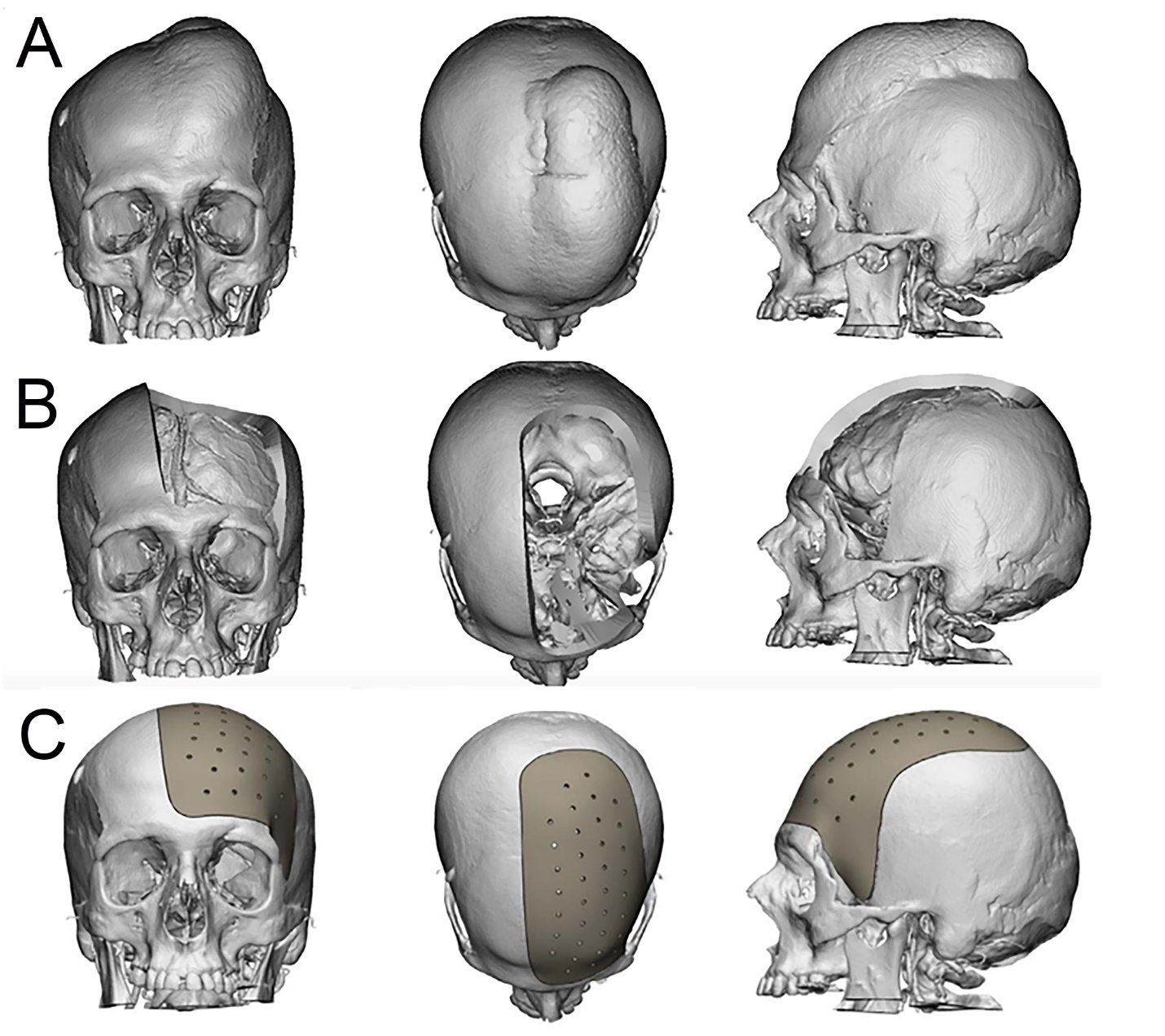


Supplemental Figure 1: Preoperative 3D CT reconstructions (A) demonstrating the large calvarial mass. Simulated area of mass resected with predicted skull defect (B). Patient-specific PEEK custom implant for single stage reconstruction of skull defect (C)
